# Predictors of Counselling Related to a Healthy Lifestyle Carried Out by a General Practitioner

**DOI:** 10.3390/ijerph16224475

**Published:** 2019-11-14

**Authors:** Małgorzata Znyk, Kinga Polańska, Piotr Wojtysiak, Michał Szulc, Leokadia Bąk-Romaniszyn, Teresa Makowiec-Dąbrowska, Justyna Zajdel-Całkowska, Dorota Kaleta

**Affiliations:** 1 Department of Hygiene and Epidemiology, Medical University of Lodz, 90-647 Lodz, Poland; kinga.polanska@umed.lodz.pl (K.P.); piotr.wojtysiak@umed.lodz.pl (P.W.); michal.szulc@imp.lodz.pl (M.S.); dkaleta@op.pl (D.K.); 2 Department of Nutrition in Digestive Tract Diseases, Medical University of Lodz, 93-338 Lodz, Poland; Leokadia.bak-romaniszyn@umed.lodz.pl; 3 Department of Work Physiology and Ergonomics, Nofer Institute of Occupational Medicine, 91-348 Lodz, Poland; Teresa.Makowiec-Dabrowska@imp.lodz.pl; 4 Faculty of Law and Administration, Lazarski University in Warsaw, 02-662 Warsaw, Poland; justyna.zajdel@umed.lodz.pl

**Keywords:** health education, primary care, general practitioner, chronic diseases, healthy lifestyle, health behaviors

## Abstract

The aim of the study was to assess whether general practitioners (GPs) monitor and evaluate the health behavior of their patients in the field of a diet, physical activity, and weight control, and whether they provide appropriate counselling as part of this evaluation. Predictors of those activities among physicians were also determined. The cross-sectional study was conducted in the Piotrkowski district among 200 GPs. The questionnaire covered socio-demographic data and lifestyle characteristics of the physicians, their role as healthy lifestyle providers, and whether they assess lifestyle characteristics of their patients and perform healthy lifestyle counselling. More than 60% of the GPs did not evaluate lifestyle features during their patients’ examination. In total, 56% of the GPs provided healthy lifestyle recommendations among patients who have not been diagnosed with chronic lifestyle-related diseases but who do not follow healthy recommendations, and 73% of GPs provided recommendations to patients with chronic diseases related to lifestyle. The study showed that the chance to assess lifestyle characteristics of the patients was significantly higher for the GPs who believed that they were obliged to do so (OR = 6.5; *p* = 0.002). The chance to recommend a healthy lifestyle among patients who have not been diagnosed with chronic lifestyle-related diseases but who do not follow healthy recommendations was 5.9 times higher among the GPs working in the public sector (*p* < 0.001) and 16.3 times higher for these who believed that they had sufficient knowledge to provide the advice (*p* = 0.02). The following predictors of providing a healthy lifestyle counselling among patients with diagnosed chronic lifestyle-related diseases were identified: conviction that a GPs is obligated to provide it (OR = 4.4; *p* = 0.02), sufficient knowledge (OR = 8.7; *p* = 0.01), and following health recommendations by themselves (OR = 3.9; *p* = 0.04). Conclusions: The identified predictors are crucial for the development of appropriate strategies aiming at increasing GPs’ involvement in preventive measures and consequently at improving the population’s health.

## 1. Introduction

Chronic diseases constitute the leading cause of death in developed countries among both men and women. They reduce a patient’s well-being and activity contributing to a poor quality of life, disability, and decreased productivity [1,2]. The risk of chronic diseases increases along with age (72% of all disorders are found among people over 30 years of age). However, the disease, when undetectable, has usually started during youth/middle age. What is more, a diagnosis that occurs at an advanced stage of the disease development results in a need for long treatment, care, and rehabilitation of patients.

According to the World Health Organization (WHO), chronic diseases contribute to the deaths of over 36 out of 57 million people worldwide each year. In 2015 they were a cause of 70% of deaths, including 37% of deaths in low-income countries and 88% of deaths in high-income countries [3]. It is estimated that chronic diseases occur in 133 million people around the world, and that this number is increasing by 1% per year, giving a population of 177 million chronically ill people in 2030. About 75% of the general population has at least one chronic disease and almost half of people with chronic illnesses have at least two diseases requiring constant contact with a health care provider [2,4]. The phenomenon of multiple diseases is most commonly observed in the elderly.

The most common chronic diseases include: cardiovascular diseases (responsible for 17.3 million deaths per year), cancer (7.6 million), chronic respiratory diseases (4.2 million), and diabetes (1.3 million) [5]. There is a possibility to prevent and delay the time of chronic diseases occurrence, among others through lifestyle modifications carried out as part of the primary and secondary prevention strategies. It is enough to eliminate risk factors such as an unhealthy diet, physical inactivity, or smoking to prevent 75% of possible occurrences of heart diseases, type 2 diabetes, and strokes, and to reduce the risk of cancer by 40% [6].

One of the main objectives of family medicine is the dominance of preventive actions over corrective medicine. Primary health care plays a key role in the healthcare system. The effectiveness of the entire medical care system depends on it, including prevention and health promotion of non-communicable diseases (NCD) [7].

Primary health care entities are a patient’s first contact with health care, therefore, they are where health education should be conducted—which is so important in prevention and promotion of health, as well as having a decisive impact on the success of using restorative medicine.

Health education as a process consisting in developing skills or acquiring knowledge in the field of health protection, aims at changing specific behaviors. It is directed not only at providing necessary information, but also at increasing motivation or serving to increase self-confidence in a patient. A patient who is properly educated will participate in the prevention and treatment process more effectively [8].

According to the assumptions of primary health care, the task of medical workers is not only to treat diseases, but first and foremost to care for the health needs of patients and to build their health potential. This model does not only apply in the USA but also in European countries, including Poland [9,10]. Polish regulations in the field of activities aimed at maintaining health of a recipient mention, among others, that a primary health care practitioner conducts health education and systematic, periodic health assessments as part of balance sheet tests. In the scope of activities, the objective of which is prevention of diseases, it is a GP who identifies risk factors for the patients’ health and undertakes actions aiming at their reduction. As part of a disease treatment, a doctor agrees on and plans educational activities with a patient so as to eliminate or reduce the condition that causes the disease [11].

Patients who make a choice and sign up for a GP usually use the services for many years, which allows for creating trust and a patient-doctor relationship, and thus, translates into improved cooperation in the process of prevention and treatment [12,13,14].

Physicians should not only focus on clinical treatment of the existing disease, but also should take appropriate steps to motivate patients to change their health behaviors. These include recognition of the health needs of their patients through active discussion, offering lifestyle counselling, pointing to positive health behaviors, and providing a patient with support and monitoring of the changes [15]. Repetitive and intensive advice from GPs can influence patients’ decisions on lifestyle modification including quitting smoking, increasing the level of leisure-time physical activity (LTPA), and translating healthier dietary patterns [15,16,17,18].

GPs consider health promotion and chronic disease prevention as important aspects of their work [19,20]. However, they notice obstacles in their implementation such as lack of time and insufficient financial support [21]. What is more, there is a growing concern among GPs that preventive measures may not provide the expected benefits [22].

The aim of the study was to assess whether GPs in Piotrkowski district monitor and evaluate health behaviors of their patients in the field of a diet, physical activity and weight control, and whether they provide appropriate counselling with this regard. The predictors of the activities undertaken by the physicians were also determined. This information is crucial for appropriate recognition of the situation, identification of the relevant needs, and the development of appropriate strategies to increase the GPs’ involvement in preventive measures and consequently to improve population health.

## 2. Material and Methods

### 2.1. Study Design and Population

This cross-sectional study was conducted among the GPs employed in primary health care for adults in Piotrkowski district. Piotrkowski district is a socially disadvantaged rural area in Lodzkie voivodship (central Poland). A detailed description of the district and the population has been published previously [23]. Briefly, in 2017 more than 90% of the residents of the district were people who lived in a rural area (villages with fewer than ten thousands of residents). What is more, the unemployment rate for this district was much higher than the national unemployment rate. It also has to be emphasized that the rate of unemployment, in the context of the rural nature of the district, is underestimated as a social problem. Piotrkowski district was also recognized as one with the lowest indicators of social development (including Health Index, Education Index, Welfare Index) compared to other rural districts in Poland.

According to the data received from the National Health Fund, there were 316 primary care physicians working in Piotrkowski district. We were able to contact 300 of them (95%). Of this group, 200 GPs agreed to participate in the study (67%). The characteristics of the GPs who agreed and who did not agree to fill in the questionnaire (with respect to gender, age, and the main place of their medical practice) have not differed significantly (Table S1).

The study was approved by the Bioethics Committee of Medical University in Lodz (RNN/315/18/KE) and GPs gave written consent to participate in the study.

### 2.2. Study Variables

The study participants filled in an anonymous, self-administered questionnaire divided into 3 sections. The first part covered socio-demographic data (age, gender), information regarding the main place of medical practice (categorized as public and private), lifestyle characteristics of the GPs (smoking status, alcohol, fruit and vegetable consumption, height and weight, LTPA), and their subjective perception of a health status (very poor/poor/good/very good). The second part of the questionnaire covered information about a physician’s role as a healthy lifestyle provider. Finally, the GPs were asked if they were evaluating lifestyle characteristics of their patients and if they had been performing health lifestyle counselling among them.

#### 2.2.1. Physicians’ Healthy Lifestyle Index

Five lifestyle characteristics were analyzed: smoking status, alcohol consumption, fruit and vegetable intake, body mass index (BMI), and LTPA. The pattern of all five lifestyle characteristics was defined as a single healthy lifestyle indicator (HLI) coded as 1 if a GP was following healthy lifestyle recommendations and as 0 if the recommendations were not followed. The lifestyle characteristics were added up to create a healthy lifestyle index (range, 0–5).

The current smoking status was evaluated based on a question “Are you currently smoking cigarettes?” (yes: HLI = 0; no: HLI = 1).

For alcohol consumption, information regarding the frequency, intensity, and type of consumed alcohol was collected. It is recommended that adults consume no more than a moderate amount of alcohol defined as up to one drink per day for women and up to two drinks per day for men [24]. It is also underlined that frequent and high-quantity alcohol consumption is generally related to a poorer health-related quality of life. HLI equal to 1 is dedicated to the participants who followed up on the recommendations.

The study subjects indicated the frequency of fruit and vegetable consumption. It is recommended that adults consume an average of eight to ten combined servings of fruit and vegetables each day [24]. The participants who consumed eight or more combined servings per day received a value of 1 for HLI.

Based on height (m) and weight (kg), BMI (kg/m^2^) was calculated and categorized following the existing guidelines [25]. Healthy weight was defined as BMI between 18.5 and 24.9 (kg/m^2^) (HLI = 1).

The participants were asked to indicate frequency (per week), intensity (moderate-intensity physical activity and vigorous-intensity physical activity) and length (minutes per day) of LTPA. It is recommended that adults participate in 150 min per week of moderate-intensity physical activity, 75 min per week of vigorous-intensity physical activity, or 115 min of combined-intensity physical activity per week [26]. The participants who achieved at least one of the above mentioned criteria for the recommended level of LTPA received HLI = 1.

#### 2.2.2. The Role of a Physician as a Healthy Lifestyle Provider

The questionnaire included the following statements regarding a physician’s role as a healthy lifestyle provider: “A physician is obligated to provide the counselling focusing on healthy lifestyle among the patients”, “I have sufficient knowledge to provide healthy lifestyle counseling for patients” and “Healthy lifestyle counselling will be more effective if a physician is following health recommendations him/herself”. For each statement five answers were possible: “strongly agree”, “rather agree”, “I do not know”, “rather disagree”, and “strongly disagree”. The first two options were considered positive (“yes”) and the other ones were considered to be negative (“no”).

Finally, the study subjects were asked if they had been evaluating lifestyle characteristic (diet, LTPA, and BMI) of their patients and if they had been performing health lifestyle counselling (focusing on a healthy diet, recommended level of LTPA, and BMI) among them.

### 2.3. Statistical Analysis

Elements of the descriptive statistics and logistic regression analysis were used to describe the obtained results. For the variables included in the study, the percentage share of individual categories of responses in the study group was calculated.

The unadjusted and adjusted odds ratios (OR) and 95% confidence intervals (95% CI) were calculated to identify the predictors of preventive measures performed by a GP. Multivariate models included all of the associations at significance level *p* = 0.1 in univariate analyses. The results for which the values of the statistics obtained in the tests used belonged to the critical area of the relevant distribution at the significance level *p* = 0.05 were considered significant.

STATISTICA version 10.0 (Dell Software, Arizona, CA, USA) was used to perform the statistical analysis.

## 3. Results

### 3.1. Characteristics of the Study Population

The characteristics of the GPs involved in the study is presented in Table 1. About 58% of the participants were male and 61% of the respondents were GPs over 40 years of age. GPs working in private health care entities constituted 148 out of 200 respondents (74%).

Thirty of the 200 GPs responded positively to the question “Do you currently smoke? “(HLI = 0). The rest were categorized as following recommendations (HLI = 1). About 70% of the participants complied with the recommendations on alcohol consumption (HLI = 1). Among the surveyed GPs, there was low consumption of vegetables and fruits; only 1/5 of them declared consumption of eight or more combined portions (HLI = 1). The recommended range of BMI (18.5–24.9 kg/m^2^) was reported by 56% of the examined GPs (they were categorized as following healthy lifestyle guidelines in this regard; HLI = 1), whereas 36% were overweight and 7% were obese. Sixty nine percent of the GPs achieved the recommended LTPA level (HLI = 1).

The healthy lifestyle index (summed up lifestyle features) between 0 to 3 was reported by 70% of the GPs and only 20% gained 4 out of 5 points. Eleven percent of the participants received the maximum number of points (indicating that they follow up all 5 recommendations related to the lifestyle). Only 12% of the respondents described their health status as very good or good.

### 3.2. The Role of a Physician as a Healthy Lifestyle Provider

Table 2 presents the possible opinions about the role of a GP as a healthy lifestyle provider. Sixty four percent of the GPs believed that the doctor was obliged to provide patients with healthy lifestyle counselling. Only 30% of the respondents thought that they had sufficient knowledge to do so. More than half of the GPs believed that healthy lifestyle counselling would be more effective if a GP him/herself followed the health recommendations (56%).

More than 60% of the GPs did not study lifestyle features (diet, LTPA, BMI) during patients’ examinations (Table 3). Fifty six percent of the GPs provided healthy lifestyle recommendations (for a diet, LTPA, and BMI) among patients who have not been diagnosed with chronic lifestyle-related diseases but who did not follow healthy recommendations, and 73% did the same among patients with chronic diseases related to lifestyle.

Table 4 shows the chance of evaluating lifestyle characteristics (a diet, LTPA, and BMI) during patients’ examinations depending on selected characteristics of a GP. The results of the univariate and multivariate logistic models are presented.

In the multivariable model, it was shown that a GP’s age remains a statistically significant predictor of lifestyle characteristics evaluation during a patient’s examination (OR = 1.1; *p* = 0.005)

Chance to assess lifestyle characteristics (a diet, LTPA and BMI) during patients’ examinations was 6.5 times greater for the GPs who believed that a doctor was required to provide patients with healthy lifestyle counselling (*p* = 0.002).

Table 5 and Table 6 show the chance of GPs providing healthy lifestyle recommendations (for a diet, LTPA, and BMI) depending on selected characteristics of that GP. The chance of recommending a healthy lifestyle among patients who have not been diagnosed with chronic lifestyle-related diseases but who did not follow healthy recommendations was 5.9 times higher among the GPs who worked in the public sector than for those working for a private company (*p* < 0.001). A significantly greater chance of recommending a healthy lifestyle among patients who have not been diagnosed with chronic lifestyle-related diseases but who did not follow healthy recommendations was reported in the case of the GPs who believed they had sufficient knowledge to provide patients with healthy lifestyle advice (OR = 16.3 *p* = 0.02).

The chance of GPs recommending a healthy lifestyle among patients with diagnosed chronic lifestyle-related diseases was 4.4 (*p* = 0.02) times higher for the GPs who thought that a GP was obliged to provide patients with health-focused counselling, 8.7 times higher for the GPs who thought that they had sufficient knowledge to provide patients with healthy lifestyle advice (*p* = 0.01), and 3.9 times higher if a GP was following health recommendations (*p* = 0.04).

## 4. Discussion

This study provides an analysis of the prevalence and predictors of healthy lifestyle counselling among adult patients by the GPs practicing in Piotrkowski district. A GP is often the only line of contact between a patient and the healthcare system in the prevention of chronic diseases. The identified predictors of counselling related to a healthy lifestyle (such as GPs knowledge, obligations, and attitudes) are crucial for the development of appropriate strategies aiming at improvement of the population’s health.

Many studies have shown that lifestyle factors such as: exercise, healthy dietary patterns, non-smoking status, recommended range of BMI, and non or moderate alcohol consumption can have a positive effect on health and quality of life [27,28,29,30,31]. GPs should be leaders with that regard. Unfortunately, the analysis of the healthy lifestyle index (summed up lifestyle characteristics) in our study showed that only 11% of the GPs fulfilled the criteria for all five evaluated healthy lifestyle factors. Earlier studies conducted by Kaleta et al. have shown that even lower percentages of the participants from the group of full-time employees (2.1% of men and 1.1% of women) met the criteria of a healthy lifestyle [32]. Similarly, Reeves et al. have indicated that the overall prevalence of the healthy lifestyle indicator was only 3% among adults in the US [33]

In our study 69% of the GPs achieved the recommended level of LTPA. Similar results were observed in a study among physicians from Poznan and Bydgoszcz (Poland), where 70% of them declared a satisfactory frequency of participation in sports and recreation classes, which was twice a week on average [34]. The results from another study conducted among physicians in Poland indicates that only 37% of respondents declared that they undertook recreational physical activity at least several times a week. Most respondents (45% of women and 1/3 of men) spent 1–2 h a week on recreational physical activity. In addition, 32.7% of men declared carrying out physical recreation for 3–4 h a week, while 14.5% of women made a similar claim [35]. Inadequate LTPA among physicians has also been described in an earlier study performed in Poland [36]. The study by Kaleta et al. illustrated a much worse situation for physical activity patterns among adults in an urban population in Poland comparing to the one observed in our study. Only 16.0% of men and 4.3% of women conducted free time physical activity at a level that provides health benefits [37]. The problem seems to mostly be a sedentary lifestyle and an unsatisfactory level of activity in leisure time. The results of the study evaluating physical activity among inhabitants of six European countries underline the gaps between Eastern and Western populations [38]. The recent data from the Ministry of Sport and Tourism of Poland indicate that about 21.8% of Poles aged 15–69 meet the standards for the level of LTPA recommended by WHO [39]. The differences between the studies can result from characteristics of the population, the year when the study was conducted, and the definition of a satisfactory/recommended level of LTPA.

Rational nutrition, including energy balance and a regular physical activity, help maintain a healthy body weight. Studies show that 58.7% Europeans are overweight and 23.33% of them suffer from obesity. Over the past 40 years, the prevalence of obesity in the world has increased almost threefold: from 6.4% to 14.9% among women and from 3.2% to 10.8% among men. If current trends persist, in 2025 21% of women and 18% of men in the world will be obese [40,41].

Kaleta et al. have shown that 24.7% women and 54.0% men were overweight, 7.5% women and 17.0% were obese [32]. In the Gacek study involving a group of doctors, the average BMI was 23.8 kg/m^2^ in women and 27.0 kg/m^2^ in men [35]. In that study the distribution of BMI was as follow: 20.5% of women and 62.5% of men were overweight, while 15% of men and women were obese. An earlier research by Bąk and Kopczynska-Sikorska on the nutritional status of medical staff has shown that 64% of physicians had a normal body weight, and 6% were overweight [36]. In our study, 36% of the GPs were overweight and 7% of them were obese.

Smoking has been decreasing in recent years in Europe. However, the study by Arvayeva et al. assessing the prevalence of smoking among GPs in Ukraine shows a high percentage of smokers (57% among men and 15% among women) [42]. In contrast, a study of GPs in Flanders has shown that smoking is rare among doctors, as opposed to alcohol consumption [43]. Our study in the medical population showed the lower prevalence of smoking among primary care physicians compared to the general population [44].

Reeves et al. have shown that only 23.3% of people consume the recommended amounts of vegetables and fruit [33]. Research by Kaleta et al. proves that 24.5% of men and 37.6% of women declared a fiber intake of 30 g/day [32]. The results of the research by Gacek have shown insufficient consumption of fruit and vegetables in the group of doctors. Doctors most often consumed vegetables once a day (this applies to 3/4 women and over half of men) [35]. The frequency of fruit consumption was similar. Our study confirms that only 20% of the GPs consume vegetables and fruit at a recommended level.

Relationships have been found between the personal health behavior of European doctors and their attitudes towards health promotion. Physicians who did physical activity felt that they were more effective in helping patients to undertake a regular physical exercise than doctors with a more sedentary lifestyle (59.14% compared with 49.70%). Doctors who smoked felt less effective at helping patients reduce tobacco consumption than the non-smoking GPs (39.34% versus 48.18%) [21].

Healthcare professionals should seek to implement healthy lifestyle activities among their patients. The results of our study show that 64% of the GPs think they are required to provide patients with healthy lifestyle counselling. Only 30% of them think they have sufficient knowledge for this.

Both the GPs and patients encounter difficulties in the sustainable and effective implementation of healthy lifestyle activities. Data on smoking cessation and smoking cessation interventions among doctors in Eastern and Central Europe are very limited. Research by Jankowski et al. has shown that doctors in Poland do not routinely intervene in the case of their patients. The study revealed that while 37.6% of physicians regularly offered smokers a minimum intervention to stop smoking, only 5% of physicians offered referrals to smoking cessation clinics [45].

A study in the US also found low frequencies of counselling on alcohol [46]. McAvoy et al. have shown that primary care physicians in the UK claimed that the greatest need for better counselling performance was related to alcohol consumption [47]. The same conclusion was reached in a Swedish study [13].

A study by Buczkowski et al. has shown how important the example of a GP is to people who are considering quitting smoking [48]. Most participants admitted that they would be more motivated to quit smoking if their GP was a non-smoker.

More than half of the GPs in our study believed that healthy lifestyle tips would be more effective if the doctor followed the health recommendations themselves (56%). Sixty three percent of the study participants did not assess lifestyle features (diet, LTPA, BMI) when examining patients. These results shows that patients do not receive the right amount of healthy lifestyle advice.

Currently, a conscious patient expects a relationship with a GP and wants to make treatment decisions together with him/her. A lack of proper interpersonal communication discredits the authority of a doctor. In the study by Nowakowska et al. 52% of respondents believed that a doctor did not devote enough time to provide the necessary information about their health [49]. During a visit he/she spent most of the available time on completing the documentation, at the expense of the time he/she could spend on counselling.

As some previous studies show, the main reasons for poor lifestyle interventions include insufficient time and lack of reimbursement for undertaking such activities [50]. A similar situation occurs in Poland, where most financial resources are spent on treatment, not on prophylaxis.

In the Jerdéna study carried out in Sweden and the US, the proportion of people declaring their willingness to get help from primary care in both countries was generally over 80%. The percentage of US patients who reported that the doctor had initiated a discussion on lifestyle modifications was, with the exception of alcohol, approximately twice the level reported by Swedish patients [10]. The EUROPREVIEW study (conducted in 22 European countries) shows that there are patient expectations regarding counselling. About half of patients who smoked, had unhealthy eating habits, or a lack of physical activity wanted their GP to provide them with advice on their habits. In addition, the study has shown that about half of patients of primary care reported that they had no discussions with their GPs about their lifestyle [51].

GPs themselves signal the need for a greater share of primary healthcare in health education in the field of healthy living [52]. Research results of Stefanowicz et al. show that doctors are aware of the leading role of primary care in health education [53]. Marcinkowski’s research conducted among primary care patients has shown that patients want GPs to promote a healthy lifestyle to them [54]. However, in another study conducted by Kulczycka et al., 83.8% of patients indicated that they obtained information about a healthy lifestyle from their GPs [55].

Pollak et al. indicate that preventive visits were longer than chronic care visits and that physicians were spending less time than recommended for important services, especially smoking cessation, Pap smear, and nutrition counseling. In the Marcinowicz study, patients found that GPs most often discussed the problems of nutrition and physical activity, while drinking alcohol and smoking were rarely mentioned [56]. There were no significant differences in the frequency of health promotion activities between the GPs in public and private healthcare facilities. 

This study was the first one evaluating whether GPs in Piotrkowski district, which is a socially disadvantaged rural area in Poland, monitor and evaluate the health behavior of their patients and whether they provide appropriate counselling with this regard. As was mentioned above, this district is recognized as one with the lowest indicators of social development (including the Health Index, Education Index, and Welfare Index) compared to other rural districts in Poland. Thus, the role of the GP not only in the treatment of the disease but also in disease prevention is crucial for improving the situation. The study evaluated a variety of factors (including the lifestyle index) that might influence GPs attitudes towards the lifestyle characteristics of their patients. Identified predictors (and limitations) of GP’s preventive activities needs to considered when developing appropriate strategies.

The main limitation of the study is related to the fact that all of the assessments were done only among the GPs and do not take into account the opinion of patients with this regards. What is more, the dependent variables (if the GPs are evaluating the lifestyle characteristic of the patients and if they are providing healthy lifestyle counselling among them) were assessed in a subjective manner (by self-reporting) that might have resulted in bias towards a virtuous response to the questions. It also needs to be pointed out that the observed patterns can reflect the situation in a disadvantaged, rural area and do not apply to the whole country. However, physicians are following the same education and their attitudes towards treatment, risk factor assessment, and counselling should be independent of where they work. Finally, even though we have evaluated a variety of factors which can determine the GP’s decisions relating to preventive activities, we have not looked at other potential determinants including limited time and financial resources.

## 5. Conclusions

Developing healthy lifestyle attitudes and raising health awareness among patients should play an important role in primary healthcare. GPs should identify health needs of the patients via an active discussion and then they should provide them with support and offer lifestyle advice.

If counselling related to a healthy lifestyle is conducted at an appropriate level, it can considerably improve the health of the population. The multifactorial model of our study showed that the chance of assessing lifestyle characteristics during examinations of patients and providing healthy lifestyle counselling among them was significantly greater for the GPs who believed that a doctor was obligated to do so and who had sufficient knowledge to provide patients with advice. Additionally, counselling and interventions carried out by GPs would be more effective if doctors themselves followed the same health recommendations. Thus, strengthening the sense of responsibility of doctors for the health of the population not only through treatment of specific diseases, but also by using preventive measures is crucial. This can be done during the medical curriculum, by performing additional courses/trainings and campaigns that aim at improving the motivation of GPs to act as healthy lifestyle providers. They should be also equipped with knowledge and tools to provide effective education and interventions among their patients. The standard operating procedures in primary healthcare (with listed institutions where the patients can receive additional support for their lifestyle modification) might be useful. Also obstacles in the implementation of preventive strategies by GPs such as lack of time and insufficient financial support need to be addressed at a country level. Finally, physicians should be role models for their patients; in order to be effective and credible in their preventive activities, they should follow healthy recommendations themselves. As indicated by our study, there is still a need for interventions designed specifically for physicians to modify their lifestyle.

## Figures and Tables

**Table 1:** Characteristics of the study participants.

Variables	*N*	%
Gender		
Female	85	42.5
Male	115	57.5
Age (years)		
≤40	78	39.0
>40	122	61.0
Medical practice		
Private	148	74.0
Public	52	26.0
Lifestyle characteristics		
Smoking healthy lifestyle indicator		
HLI = 0	30	15.0
HLI = 1	170	85.0
Alcohol healthy lifestyle indicator		
HLI = 0	61	30.5
HLI = 1	139	69.5
Combined fruit and vegetable healthy lifestyle indicator		
HLI = 0	161	80.5
HLI = 1	39	19.5
BMI		
<18.5 kg/m^2^	2	1.0
18.5–24.9 kg/m^2^	112	56.0
25.0–29.9 kg/m^2^	72	36.0
≥30.0 kg/m^2^	14	7.0
BMI healthy lifestyle indicator		
HLI = 1	112	56.0
HLI = 0	88	44.0
LTPA		
Moderate-intensity physical activity		
<150 min per week	101	50.5
≥150 min per week	99	49.5
Vigorous-intensity physical activity		
<75 min per week	156	78.0
≥75 min per week	44	22.0
Combined-intensity physical activity ^a^		
<115 min per week	63	81.8
≥115 min per week	14	18.2
LTPA healthy lifestyle indicator		
HLI = 0	63	31.5
HLI = 1	137	68.5
Healthy lifestyle index		
0	1	0.5
1	20	10.0
2	43	21.5
3	75	37.5
4	39	19.5
5	22	11.0
Health status		
Very good/good	23	11.5
Poor/very poor	177	88.5

HLI—healthy lifestyle indicator. BMI—body mass index. LTPA—leisure-time physical activity. ^a^—for the participants who did not achieve the recommended level of neither moderate—nor vigorous-intensity physical activity.

**Table 2:** Opinions about the role of a physician as a healthy lifestyle provider.

Opinion	*N*	%
A physician is obliged to provide counselling focusing on a healthy lifestyle among the patients		
Yes	137	63.5
No	63	31.5
I have sufficient knowledge to provide healthy lifestyle counselling among the patients		
Yes	59	29.5
No	141	70.5
The healthy lifestyle counselling will be more effective if a physician is following health recommendations		
Yes	111	55.5
No	89	44.5

**Table 3:** Assessment of lifestyle features (diet, LTPA and BMI) in patients.

Opinion	*N*	%
A physician is evaluating lifestyle characteristics (a diet, LTPA and BMI) during patients’ examinations		
No	126	63.0
Yes	74	37.0
A physician is providing healthy lifestyle recommendations (for a diet, recommended level of LTPA and BMI) to patients not diagnosed with chronic diseases related to lifestyle but who are not following healthy recommendations		
No	88	44.0
Yes	112	56.0
A physician is providing healthy lifestyle recommendations (for a diet, recommended level of LTPA and BMI) to patients diagnosed with chronic diseases related to lifestyle		
No	55	27.5
Yes	145	72.5

**Table 4:** Chance of evaluating lifestyle characteristics (a diet, LTPA and BMI) during patients’ examinations depending on selected characteristics of a GP.

Characteristics	Unadjusted Model		Adjusted Model	
	OR (95% CI)	*p*-Value	OR (95% CI)	*p*-Value
Gender				
Female	1.36 (0.76–2.44)	0.295		
Male	1.00			
Age (years)	1.06 (1.04–1.09)	<0.001	1.05 (1.03–1.09)	0.005
Medical practice				
Private	1.0			
Public	1.51 (0.79–2.88)	0.212		
Healthy lifestyle index				
0–3	1.0		1.0	
4–5	0.11 (0.05–0.28)	<0.001	0.67 (0.25–1.23)	0.351
Physician is obligated to provide counselling focusing on healthy lifestyle among the patients				
Yes	5.42 (2.47–11.90)	<0.001	6.47 (1.97–21.24)	0.002
No	1.0		1.0	
I have sufficient knowledge to provide healthy lifestyle counselling for patients				
Yes	2.28 (1.22–4.27)	0.010	1.34 (0.60–2.99)	0.467
No	1.0		1.0	
Healthy lifestyle counselling would be more effective if a physician is following health recommendations				
Yes	2.96 (1.59–5.49)	<0.001	1.13 (0.45–2.59)	0.920
No	1.0		1.0	

**Table 5:** Chance of providing healthy lifestyle recommendations (for a diet, recommended level of LTPA, and BMI) to patients not diagnosed with chronic diseases related to lifestyle but who are not following healthy recommendations depending on selected characteristics of a GP.

Characteristics	Unadjusted Model		Adjusted Model	
	OR (95% CI)	*p*-Value	OR (95% CI)	*p*-Value
Gender				
Female	1.22 (0.69–2.16)	0.490		
Male	1.00			
Age (years)	1.00 (0.97–1.02)	0.740		
Medical practice				
Private	1.0		1.0	
Public	5.47 (2.47–12.06)	<0.001	5.89 (2.51–13.85)	<0.001
Healthy lifestyle index				
0–3	1.0			
4–5	0.74 (0.40–1.36)	0.330		
A physician is obligated to provide counselling focusing on healthy among the patients				
Yes	1.24 (0.68–2.63)	0.486		
No	1.0			
I have sufficient knowledge to provide healthy lifestyle counselling among the patients				
Yes	15.45 (5.78–41.35)	<0.001	16.26 (5.95–44.49)	0.024
No	1.0		1.0	
The healthy lifestyle counselling will be more effective if a physician is following health recommendations				
Yes	1.07 (0.61–1.89)	0.810		
No	1.0			

**Table 6:** Chance of providing healthy lifestyle recommendations (for a diet, recommended level of LTPA, and BMI) to patients diagnosed with chronic diseases related to lifestyle depending on selected characteristics of a GP.

Characteristics	Unadjusted Model		Adjusted Model	
	OR (95% CI)	*p*-Value	OR (95% CI)	*p*-Value
Gender				
Female	0.62 (0.33–1.17)	0.142		
Male	1.0			
Age (years)	1.00 (0.97–1.03)	0.960		
Medical practice				
Private	1.0			
ublic	0.50 (0.25–0.97)	0.043	1.05 (0.41–2.72)	0.917
Healthy lifestyle index				
0–3	1.0			
4–5	0.55 (0.29–1.06)	0.080	0.59 (0.21–1.67)	0.301
A physician is obligated to provide counselling focused on healthy lifestyle among the patients				
Yes	16.37 (7.62–35.16)	<0.001	4.38 (1.31–14.68)	0.017
No	1.0		1.0	
I have sufficient knowledge to provide healthy lifestyle counselling among the patients				
Yes	17.16 (3.97–74.18)	<0.001	8.67 (1.78–42.29)	0.008
No	1.0		1.0	
The healthy lifestyle counselling will be more effective if a physician is following health recommendations				
Yes	12.12 (5.42–27.12)	<0.001	3.91 (1.08–14.10)	0.038
No	1.0		1.0	

## Supplementary Materials

The following are available online at https://www.mdpi.com/1660-4601/16/22/4475/s1. Table S1. Characteristics of the GPs who agreed and refused to participate in the study (percentages were shown).
