# Supplementary material for: Predictors of Counselling Related to a Healthy Lifestyle Carried Out by a General Practitioner
Source: Int J Environ Res Public Health. 2019 Nov 14;16(22):4475. doi: 10.3390/ijerph16224475 (PMC6888170; doi:10.3390/ijerph16224475)
Supplement: Supplementary file 1 [file ijerph-16-04475-s001.pdf]

**Table S1.** Characteristics of the GPs who agreed and refused to participate in the study (percentages were shown).

| Variables | GPs Who Agreed to Participate in the Study (*N* = 200) | GPs Who Refused to Participate in the Study (*N* = 100) | *p* |
|---|---|---|---|
| | Gender | | |
| Female | 42.5 | 40.0 | 0.77 |
| Male | 57.5 | 60.0 | |
| | Age (years) | | |
| ≤40 | 39.0 | 41.0 | 0.83 |
| >40 | 61.0 | 59.0 | |
| Medical practice | | | |
| Private | 74.0 | 75.0 | |
| Public | 26.0 | 25.0 | 0.97 |
